# Investigation Tracing the Origin of Tan Sheep Visceral Tissues through Mineral Elements

**DOI:** 10.3390/foods12132438

**Published:** 2023-06-21

**Authors:** Yanru Hou, Xuerong Wang, Dongsong Yang, Yulong Luo, Yalei Li, Ruiming Luo

**Affiliations:** College of Food Science and Engineering, Ningxia University, Yinchuan 750021, China

**Keywords:** Tan sheep, visceral tissues, mineral element, geographical traceability

## Abstract

The traceability of quality mineral fingerprints in the viscera of Tan sheep from northwest China was studied. Twenty-five mineral elements in the heart and liver samples of Tan sheep were determined using an inductively coupled plasma mass spectrometer (ICP-MS), and the characteristics of the mineral elements in the visceral tissues of the Tan sheep were further analyzed in combination with a principal component analysis (PCA), hierarchical cluster analysis (HCA), and linear discriminant analysis (LDA) to establish a discriminant model and verify it. The results show that 11 elements (^137^Ba, ^43^Ca, ^63^Cu, ^56^Fe, ^39^K, ^31^P, ^60^Ni, ^78^Se, ^118^Sn, ^125^Te, and ^66^Zn) in the Tan sheep heart samples had significant differences among different regions (*p* < 0.05), and the results of the LDA show that the accuracy rate of the return-generation examination was 85.70%, and the accuracy rate of the hand-over-fork examination was 87.50%; 10 elements (^111^Cd, ^59^Co, ^52^Cr, ^56^Fe, ^39^K, ^55^Mn, ^95^Mo, ^23^Na, ^121^Sb, and ^78^Se) in the Tan sheep liver samples had significant differences among different regions (*p* < 0.05), and the results of the LDA showed that the accuracy rate of the return-generation examination was 96.30%, and the accuracy rate of the hand-over-fork examination was 86.25%. This indicates that the multi-element analysis has potential for determining the origin of Tan sheep viscera in certain regions.

## 1. Introduction

With the development of economic globalization, the cross-border and cross-regional food trade is increasing day by day, and food safety incidents occur frequently [1]. With growing concerns about food quality, consumers are increasingly aware of the need for food certification and origin traceability. Food traceability and authenticity discrimination facilitate the implementation of origin protection, especially for regionally known brands [2].

The definition of “meat” also includes offal, which is an important dietary component that contains high-quality protein, lipids, fat-soluble vitamins, B vitamins, and minerals and is the main source of protein in food [3]. The content of these nutrients largely determines the taste and edible value of meat. Offal is a very important by-product of sheep slaughtering and processing, especially in the heart and liver. Biel et al. [4] have shown that almost all by-products (the liver, heart, kidney, and brain) of veal, beef, and lamb are rich sources of trace elements, which are usually found at much higher levels in offal than in the semitendinosus. Overall, animal visceral tissues are a valuable resource with high nutritional value. Meat consumption is projected to remain high in developed nations over the next decade, while the demand for meat in developing countries across Asia and Latin America is expected to quadruple [5]. The increase in global meat consumption can be attributed to the globalization of food systems [6], demographic shifts [7], and a growing demand for animal-protein-rich foods in certain developing nations due to nutritional requirements [8]. The increasing consumption of meat results in the generation of substantial quantities of animal by-products within slaughterhouses [9,10]. Recycling and utilizing edible visceral tissue from animal by-products reduce the environmental impact and processing costs in the meat industry’s supply chain. Moreover, the current trend is increasingly focused on reducing food waste and using by-products, thereby increasing the sustainability of the food industry. This constitutes a promising scenario for meat products containing offal extracts [11]. In China, visceral tissues can be processed into different delicacies, such as pork lungs in chili sauce, mutton sweetbread, and duck blood in chili sauce. In certain European countries and the southern United States, chitterlings, trotters, tongues, and other livestock offal are also common menu items. Toldra et al. [12] isolated two protein components from a pig spleen and utilized them as substitutes for functional components in cooked sausages. The findings demonstrated that the two spleen extracts exhibited excellent functionality and could serve as suitable sources of non-allergenic functional proteins in meat products. Therefore, the conversion of offal into more convenient food products or the development of novel functional ingredients for the food industry can enhance value and cater to current dietary trends [13,14]. In conclusion, it is necessary to trace the origins of animal viscera and establish a fast and efficient discrimination technique.

The Yanchi Tan sheep is a geographical landmark product of Ningxia, China, and its meat is tender, tasty, and flavorful, leading it to be loved by consumers [15]. The visceral tissues of Tan sheep may be adulterated with poor-quality or counterfeit products to gain economic benefits. Therefore, for the further development of the Tan sheep industry, it is necessary to establish an effective geographic traceability technology for the visceral tissues of Tan sheep to protect consumers’ interests and better promote the development of Tan sheep by-products.

Currently, many analytical techniques are being applied in traceability and adulteration studies, such as stable isotope technology [16,17], DNA fingerprint origin tracing technology [18,19], near-infrared spectroscopy technology [20], mineral element traceability technology [21], and gas chromatography [22]. Among these technologies, DNA fingerprinting refers to genetic markers developed based on DNA molecular markers, which are based on differences in nucleotide sequences among individuals. Few of these techniques have been studied with the use of minerals to identify the origin and authenticity of mutton. Wang et al. [23] determined the mineral element content of 128 Inner Mongolian sheep for the effective discrimination of sheep breed, origin, and feeding method. Smeti et al. [24] analyzed lambs from different regions and production systems via fatty acid profiles and stable isotope comparisons, and the results showed that different production systems and origins had strong effects on the distribution of the lambs’ fatty acids and that all isotopic analysis parameters, including δ(13C), δ(15N), δ(2H), and δ(18O), could effectively distinguish lambs from different geographical origins and production systems. However, there is no report on the traceability of Tan sheep visceral tissues in the literature.

In this study, the contents of 25 mineral elements in the offal (heart and liver) of Tan sheep from different provinces and within one province were analyzed via inductively coupled plasma mass spectrometry (ICP-MS). The aim was to explore the feasibility of using multi-element fingerprinting in combination with a multivariate data analysis to discriminate the geographic origin of Tan sheep offal at different regional scales.

## 2. Materials and Methods

### 2.1. Sample Information

**Figure 1:**
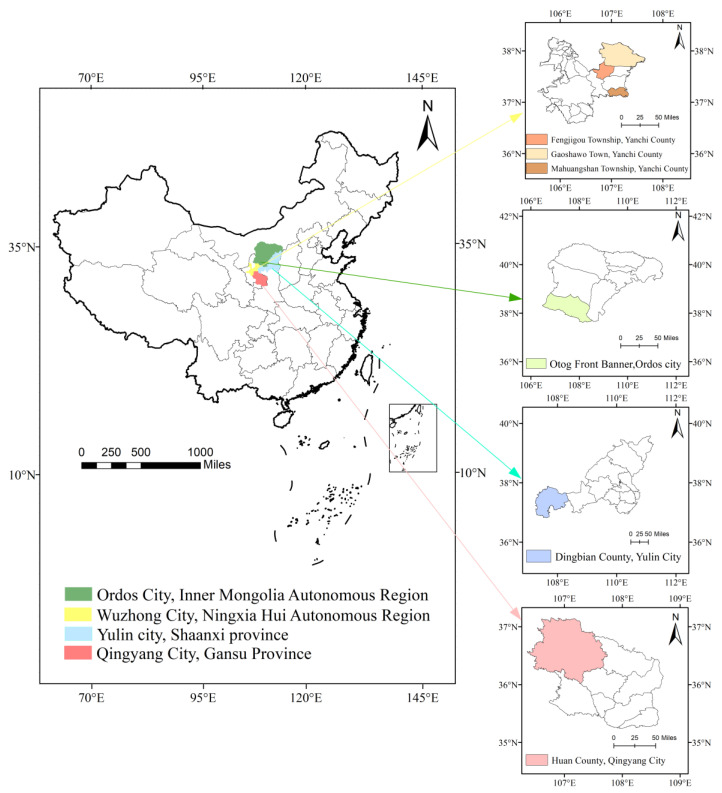
Geographical map of Tan sheep sampling sites.

For the purpose of this study, no animals were slaughtered as the offal was obtained from the slaughterhouse. Therefore, ethical approval for animal experimentation was deemed unnecessary. The processing of carcass samples strictly adhered to the Guidelines for Animal Experimentation Systems of Ningxia University. Seven-month-old healthy, castrated Tan sheep rams with an average body weight of 18.00 ± 2.00 kg were selected and fed via traditional grazing. A total of 60 heart and liver samples were collected from Yanchi County, Wuzhong City, Ningxia (*n* = 30); Etokeqian Qi, Ordos City, Inner Mongolia (*n* = 10); Dingbian County, Yulin City, Shaanxi Province (*n* = 10); and Huanxian County, Qingyang City, Gansu Province (*n* = 10). In order to obtain representative samples and determine the characteristic elements of visceral tissues of Tan sheep in Yanchi County, Ningxia, three main Tan sheep breeding villages were selected in Yanchi County, Wuzhong, Ningxia, namely, Mahuangshan Township (*n* = 10), Gaoshawo Township (*n* = 10), and Fengjiigou Township (*n* = 10). The geographical location of the samples is shown in Figure 1, and the details of all samples are shown in Table 1.

### 2.2. Sample Preparation

The heart and liver samples were precisely weighed at 100.00 g, freeze-dried for 48 h, and subsequently ground into powder form using a freeze-mill before being passed through a 200-mesh sieve. The resulting powder was stored under vacuum at −23 °C in individual bags weighing 10 g each to facilitate digestion preparation.

### 2.3. Element Analysis

#### 2.3.1. Microwave Digestion

The microwave digestion method for samples was conducted in accordance with Mi’s method [25], with minor modifications implemented to enhance precision and accuracy. The powdered samples weighing 150 mg were placed into a Teflon digestion tube, which was followed by the addition of 2.00 mL of H_2_O_2_ (30% *v*/*v*; Beijing Institute of Chemical Reagents, Beijing, China) and 6.00 mL of concentrated HNO_3_ (65% *v*/*v*; Beijing Institute of Chemical Reagents, Beijing, China) to pre-digest for 20 min. The mixture was then subjected to microwave digestion using the Mars Lp5 microwave system (CEM, Matthews, NC, USA). The digestion procedure was as follows: between 0 and 10 min, increase the temperature from 0 °C at a rate of 18 °C/min to 180 °C, and then hold for 5 min; for 10 min, increase the temperature at a rate of 6 °C/min to 240 °C, and then hold for 25 min; and finally, ventilate to drive the acid for 10 min to obtain the digestion solution. The overall digested solutions were transferred to 100 mL centrifuge tubes and subsequently diluted with distilled deionized water to a final volume of approximately 100 mL (Milli-Q Millipore, 18.2 MX/cm).

#### 2.3.2. Inductively Coupled Plasma Mass Spectrometry Determination

The content of mineral elements was measured by ICP-MS (Agilent 7500 Series ICP-MS, Agilent Technologies, Santa Clara, CA, USA). The method of ICP-MS analysis for samples was carried out according to Mi’s method (Mi, Shang, Li et al., 2019). The analysis of each sample was performed in triplicate and quantified using external standards. The Environmental Calibration Standard (Part Number 5183-4688) and the Multi-Element Calibration Standard (Part Number 8500-6944), supplied by Agilent Technologies (Palo Alto, CA, USA), were used as the standard solution and the determination coefficient of the standard curve was higher than 0.99. Internal Standard Multi-Element Mix #4 (Part Number 5190-8593), containing ^72^Ge, ^115^In, and ^209^Bi and supplied by Agilent Technologies (Palo Alto, CA, USA), was used to ensure the stability of the instrument. Samples were remeasured whenever the relative standard deviation (RSD) of internal standards was higher than 10%.

### 2.4. Statistical Analysis

The data obtained from all measurements were presented as the mean ± standard deviation (SD). Subsequently, the multi-element data were exported to R (R Core Team, 2016) for conducting a multivariate statistical analysis that included analysis of variance (ANOVA), principal component analysis (PCA), hierarchical clustering analysis (HCA), and linear discriminant analysis (LDA). The ANOVA method was utilized to analyze 25 identified minerals, with the aim of identifying significant data variables in each parameter group for discriminating geographical origins and species of visceral tissues. The significance level was set at *p* < 0.05. To assess sample similarity, HCA, an unsupervised classification method, was applied to the multi-element data.

## 3. Results

### 3.1. Analysis of Elemental Content Detection Results

The mineral element contents of 25 Tan sheep offal samples were analyzed, and the regression equations, correlation coefficients, relative standard deviation, and limits of detection for various elements are presented in Table 2. For the standard curve equations of the elements analyzed in this study, a mass concentration gradient with good fitting was selected for plotting. As shown in Table 2, the linear equation phase relationship values of ^59^Co, ^133^Cs, ^60^Ni, ^31^P, and ^121^Sb were greater than 0.9900, and the linear equation phase relationship values of the remaining 20 mineral elements were greater than 0.9990, with a relative standard deviation less than 8.0000%. During the measurement, the instrument underwent blank sample injection 8 times to calculate the detection limit of each element. The resulting range of detection limits was 0.0013~5.3420 μg/L, indicating that the measurement results could be analyzed.

### 3.2. Fingerprint Characteristics of Mineral Elements in Internal Viscus of Tan Sheep in Different Regions

#### 3.2.1. Fingerprint Characteristics of Mineral Elements in the Heart of Tan Sheep in Different Regions

From Table 3, it can be seen that the heart tissue of Tan sheep exhibited significant regional differences in the content of elements including ^137^Ba, ^43^Ca, ^63^Cu, ^56^Fe, ^39^K, ^23^Na, ^60^Ni, ^31^P, ^78^Se, ^125^Te, and ^66^Zn. Notably absent was ^107^Ag, while ^133^Cs, ^59^Co, and ^51^V were found to be uniformly distributed across all regions.

The heart tissue of Tan sheep in Yanchi County, Ningxia, exhibited the highest contents of ^52^Cr, ^39^K, ^24^Mg, ^23^Na, ^60^Ni, ^78^Se, ^118^Sn, ^125^Te, and ^66^Zn. The content of ^39^K was significantly higher than that in Shaanxi Dingbian, the contents of ^23^Na and ^118^Sn were significantly higher than that in Qianqi, Inner Mongolia, and the content of ^78^Se was significantly higher than that in the other three regions. The element content of ^137^Ba, ^9^Be, ^59^Co, ^63^Cu, and ^88^Sr was the highest in the heart tissue of Tan sheep in Otoke Qianqi, Inner Mongolia. The contents of ^43^Ca, ^44^Ca, ^111^Cd, ^85^Rb, and ^121^Sb were the highest in the heart tissue of Tan sheep in Dingbiantan, Shaanxi Province, with the content of ^43^Ca being significantly higher than that in the heart tissue of Tan sheep in Huanxian County, Gansu. The contents of ^56^Fe, ^55^Mn, ^95^Mo, and ^31^P were the highest in the heart tissue of Tan sheep in Huanxian County, Gansu. In the Huanxian County, the ^43^Ca, ^44^Ca, ^111^Cd, ^60^Ni, ^85^Rb, and ^66^Zn element content was the lowest, and the ^43^Ca element content was significantly lower than that in the heart tissue of Tan sheep in Otoke Qianqi, Inner Mongolia. The coefficient of variation is the ratio of the mean to the standard deviation and reflects the degree of dispersion of the data; the larger the value, the greater the degree of dispersion and vice versa. The coefficient of variation of ^111^Cd in Qianqi, Inner Mongolia, was 33.59%; the coefficient of variation of ^52^Cr in Yanchi County, Ningxia, was 46.26%; the coefficient of variation in Dingbian County, Shaanxi Province, was 36.73%; the coefficients of variation of the ^24^Mg and ^66^Zn elements in Huanxian County, Gansu, were 25.04% and 31.57%, respectively; and the coefficients of variation of ^59^Co, ^95^Mo, and ^118^Sn were 125%, 182%, and 130% in Yanchi County, Ningxia, respectively, exceeding 100%. The regional differences among these elements were indistinct.

#### 3.2.2. Fingerprint Characteristics of Mineral Elements in the Liver of Tan Sheep in Different Regions

According to Table 4, significant differences were observed in the levels of ^137^Ba, ^111^Cd, ^52^Cr, ^56^Fe, ^39^K, ^24^Mg, ^55^Mn, ^23^Na, ^31^P, ^78^Se, ^121^Sb, and ^88^Sr among the four regions in the liver tissue of Tan sheep.

The content of ^39^K, ^24^Mg, ^23^Na, ^60^Ni, ^31^P, ^85^Rb, and ^78^Se was the highest in the liver tissue of Tan sheep in Yanchi County, Ningxia, whereas the content of ^60^Ni was the lowest in Qianqi, Inner Mongolia, and the content of ^39^K, ^24^Mg, ^23^Na, ^31^P, and ^78^Se was the lowest in Dingbian County, Shaanxi; notably, the content of ^78^Se was significantly lower than that in other regions. The contents of the elements ^137^Ba, ^63^Cu, ^56^Fe, ^55^Mn, ^95^Mo,^121^Sb, ^88^Sr, and ^51^V were the highest in the liver tissues of Tan sheep in Huanxian County, Gansu Province, among which the contents of the elements ^137^Ba and ^88^Sr were significantly higher than those in Yanchi County, Ningxia, and the contents of the elements ^56^Fe and ^121^Sb were significantly higher than those in Qianqi, Inner Mongolia. In addition, the coefficients of variation of ^59^Co, ^60^Ni and ^51^V were 163%, 81.14%, and 100% in Yanchi County, Ningxia; 128%, 82.08%, and 200% in Otoke Qianqi, Inner Mongolia; and the coefficient of variation of ^133^Cs was 100% in Shaanxi. The coefficient of variation for these elements was found to be high in these studied regions, which may result in insignificant difference among mineral elements concentration across regions.

### 3.3. Analysis of Mineral Element Differences in Internal Visceral Tissues of Tan Sheep in Yanchi County, Ningxia

#### 3.3.1. Analysis of Mineral Element Differences in Heart Tissues of Tan Sheep in Yanchi County, Ningxia

The mineral elements contained in the heart of Tan sheep in Yanchi County, Ningxia were analyzed, as shown in Table 5, and significant differences (*p* < 0.05) were found in ^137^Ba, ^43^Ca, ^111^Cd, ^63^Cu, ^56^Fe, ^39^K, ^55^Mn, ^60^Ni, ^31^P, ^78^Se, ^118^Sn, ^125^Te and ^66^Zn (*p* < 0.05). The findings were essentially in line with the characteristic elements of the four regions heart tissues outlined in Section 3.2.1, featuring identical elements is ^137^Ba, ^43^Ca, ^63^Cu, ^56^Fe, ^60^Ni, ^31^P, ^78^Se, ^125^Te, and ^66^Zn, which could be further analyzed to determine the unique characteristic elements of the heart tissue of Yanchi Tan sheep.

#### 3.3.2. Analysis of Mineral Element Differences in Liver Tissues of Tan Sheep in Yanchi County, Ningxia

The mineral elements contained in the liver of Tan sheep in Yanchi County, Ningxia, were analyzed, as shown in Table 6, ^137^Ba, ^43^Ca, ^44^Ca, ^111^Cd, ^59^Co, ^52^Cr, ^56^Fe, ^39^K, ^55^Mn, ^95^Mo, ^23^Na, ^121^Sb, ^78^Se, ^88^Sr, ^125^Te, and ^66^Zn had significant differences among regions (*p* < 0.05). The hepatic characteristic elements were basically the same as those in the four regions, with the same elements ^111^Cd, ^56^Fe, ^39^K, ^55^Mn, ^23^Na, and ^78^Se.

### 3.4. Analysis of Mineral Element Principal Components in Internal Visceral Tissues of Tan Sheep in Yanchi County, Ningxia

#### 3.4.1. Analysis of Mineral Element Principal Components in the Heart Tissue of Tan Sheep in Yanchi County, Ningxia

**Figure 2:**
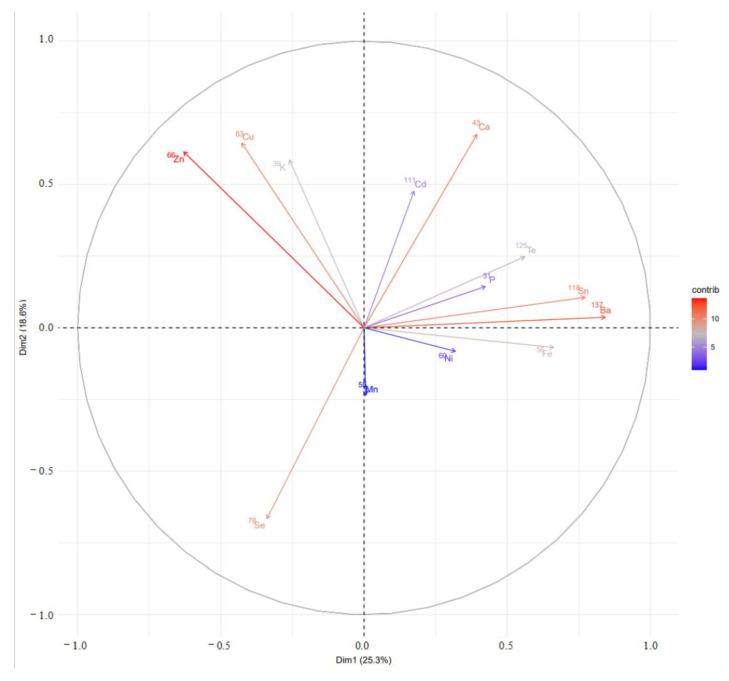
Scatter plot of PCA scores for Tan sheep heart samples.

PCA utilizes dimension reduction to analyze the internal relationships among various experimental indicators, resulting in the creation of several independent comprehensive indicators while retaining over 80% of the original information [26]. Therefore, a further analysis was conducted on the principal components of 13 mineral elements that exhibited significant differences in the heart tissue of Tan sheep in Yanchi County, Ningxia. The resulting principal component contribution load map is shown in Figure 2.

As can be observed from Figure 2, the contribution rate of the first two principal components of mineral elements exhibiting significant differences in the heart tissue of Tan sheep in Yanchi County, Ningxia, was 46.60%. The elements ^66^Zn, ^137^Ba, ^118^Sn, ^43^Ca, ^125^Te, ^78^Se, ^63^Cu, ^111^Cd, and ^56^Fe constitute the first two principal components, and are listed in descending order of contribution value. Combined with the findings from Section 3.3.1, among the 25 mineral elements measured, ^137^Ba, ^43^Ca, ^63^Cu, ^56^Fe, ^39^K, ^60^Ni, ^31^P, ^78^Se, ^118^Sn, ^125^Te, and ^66^Zn could be used as the characteristic elements of the heart tissues in Yanchi Tan sheep.

#### 3.4.2. Analysis of Mineral Element Principal Components in the Liver Tissue of Tan Sheep in Yanchi County, Ningxia

**Figure 3:**
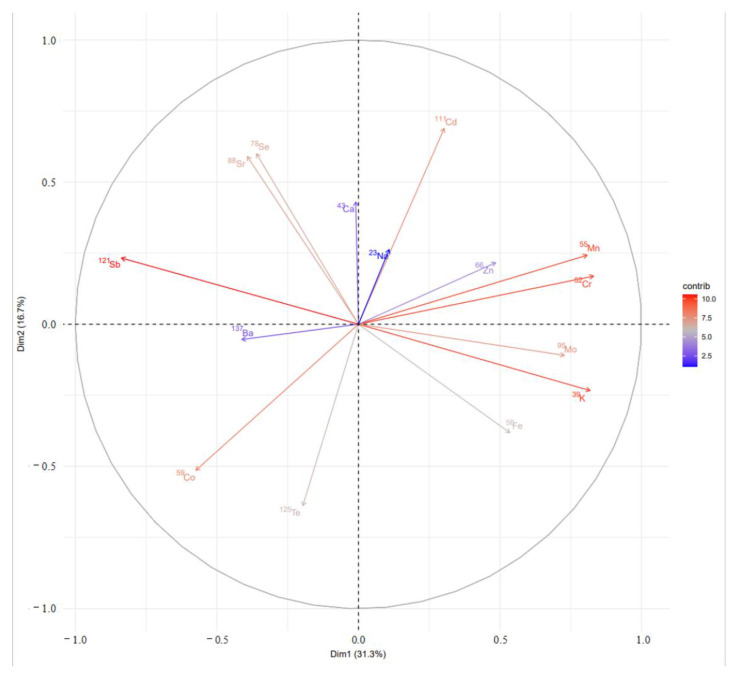
Scatter plot of PCA scores for Tan sheep liver samples.

The principal components of 15 mineral elements with significant differences in the liver of Tan sheep in Yanchi County, Ningxia, were further analyzed. The resulting principal component contribution load map is shown in Figure 3. It can be seen that the contribution rate of the first two principal components of mineral elements with significant differences in the liver tissue of Tan sheep in Ningxia Yanchi County was 48.80%. The elements ^121^Sb, ^39^K, ^55^Mn, ^52^Cr, ^59^Co, ^111^Cd, ^95^Mo, ^88^Sr, ^78^Se, ^125^Te, and ^56^Fe constitute the first two principal components, and are listed in descending order of contribution value. Combined with the findings from Section 3.3.2, among the 25 mineral elements measured, ^111^Cd, ^59^Co, ^52^Cr, ^56^Fe, ^39^K, ^55^Mn, ^95^Mo, ^23^Na, ^121^Sb, and ^78^Se could be used as characteristic elements of the liver tissue in Yanchi Tan sheep.

### 3.5. Discriminant Analysis of Mineral Elements in Visceral Tissues of Tan Sheep of Different Origin

The origin of the visceral tissues of Tan sheep was analyzed by using mineral elements (heart: ^137^Ba, ^43^Ca, ^63^Cu, ^56^Fe, ^39^K, ^31^P, ^60^Ni, ^78^Se, ^118^Sn, ^125^Te, and ^66^Zn; liver: ^111^Cd, ^59^Co, ^52^Cr, ^56^Fe, ^39^K, ^55^Mn, ^95^Mo, ^23^Na, ^121^Sb, and ^78^Se) that were closely related to the visceral tissues of Tan sheep, with reference to Qian et al. [27]. The measured samples were randomly divided into two groups, one for the training set and the other for the test set that accounted for 2/3 and 1/3 of the total sample size, respectively, to establish a discriminant model and test the effectiveness of the established discriminant model.

#### 3.5.1. Discriminant Analysis of Mineral Elements in Heart Tissues of Tan Sheep from Different Origins

The mineral elements ^137^Ba, ^43^Ca, ^63^Cu, ^56^Fe, ^39^K, ^31^P, ^60^Ni, ^78^Se, ^118^Sn, ^125^Te, and ^66^Zn, which were found to be closely related to the heart tissue of Tan sheep in Yanchi County, Ningxia, were used to establish a discriminant model, and the model coefficient results are shown in Table 7.

**Figure 4:**
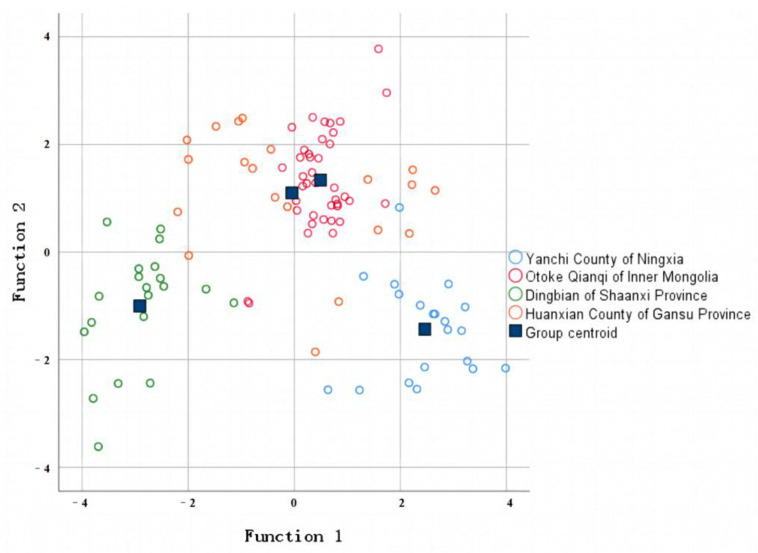
Scatter plot of Tan sheep heart samples for the first two discriminant functions.

The discriminant model incorporated ^43^Ca, ^63^Cu, ^39^K, ^31^P, ^78^Se, ^125^Te, and ^66^Zn as significant regional discriminators. The specific models for discrimination were formulated as follows:

(1) $$y_{1}=0.17x_{1}-0.97x_{2}+0.09x_{3}+0.11x_{4}+33.05x_{5}-0.90x_{6}+0.14x_{7}-161.79$$

(2) $$y_{2}=0.13x_{1}+0.02x_{2}+0.08x_{3}+0.12x_{4}+43.42x_{5}+0.05x_{6}+0.14x_{7}-158.22$$

(3) $$y_{3}=0.18x_{1}+0.27x_{2}+0.05x_{3}+0.08x_{4}+44.80x_{5}-1.31x_{6}+0.29x_{7}-124.86$$

(4) $$y_{4}=0.14x_{1}-0.08x_{2}+0.07x_{3}+0.12x_{4}+32.36x_{5}-1.09x_{6}+0.17x_{7}-144.56$$

where *y*_1_, *y*_2_, *y*_3_, and *y*_4_ represent the calculated values of the four origin models of Yanchi County, Ningxia, Otoke Qianqi, Inner Mongolia, Dingbian County, Shaanxi, and Huanxian County, Gansu, respectively. Meanwhile, *x*_1_ to *x*_7_ represent mineral element values (mg/kg) for ^43^Ca, ^63^Cu, ^39^K, ^31^P, ^78^Se, ^125^Te, and ^66^Zn. Using this discriminant model, the heart samples of the Tan sheep were classified. The measured mineral element content value was brought into the above model to calculate the corresponding *y* value; the y value was compared, and then the samples were classified as the largest category of the *y* value. Finally, the effectiveness of the established discriminant model was verified by combining the results of the return-generation examination and hand-over-fork examination. The result data are shown in Table 8, while Figure 4 shows the discriminant figure.

From Table 8, it can be seen that the overall accuracy rates of the return-generation and hand-over-fork examinations for discriminating for the heart samples of Tan sheep were 85.70% and 87.50%, respectively, indicating a superior overall discriminatory effect. In the return-generation examination, the discrimination effect of HuanXian County in Gansu Province was the best, and the sample from Yanchi County in Ningxia was the second best, but in the hand-over-fork examination, the discrimination effect of Yanchi County in Ningxia was the best. Samples from each region have been distinguished from those of other regions, which may indicate that the origin of the Tan sheep was relatively close to another region and the growth environment was similar, resulting in slight differentiation.

#### 3.5.2. Discriminant Analysis of Mineral Elements in Liver Tissues of Tan Sheep from Different Origins

The mineral elements ^111^Cd, ^59^Co, ^52^Cr, ^56^Fe, ^39^K, ^55^Mn, ^95^Mo, ^23^Na, ^121^Sb, and ^78^Se, which were closely related to the liver tissue of Tan sheep in Yanchi County, Ningxia, were used to establish a discriminant model, and the model coefficient results are shown in Table 9.

**Figure 5:**
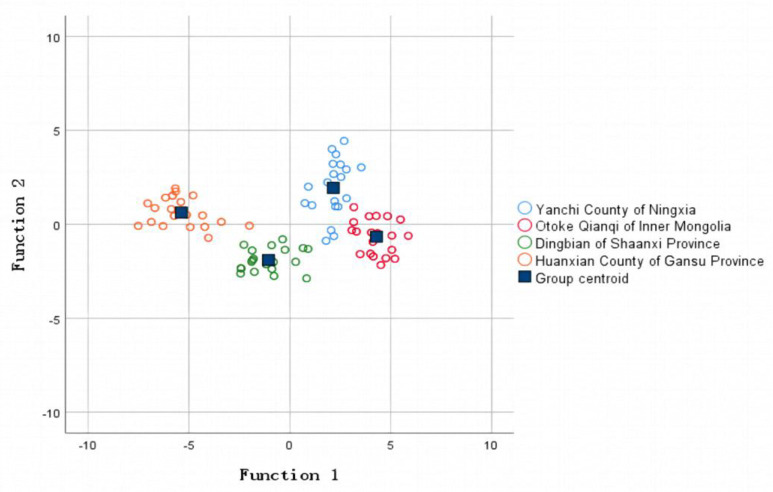
Scatter plot of Tan sheep liver samples for the first two discriminant functions.

The discriminant model incorporated ^111^Cd, ^52^Cr, ^56^Fe, ^23^Na, ^121^Sb, and ^78^Se as indicating significant regional discrimination. The specific discriminant models were formulated as follows:

(5) $$y_{1}=1.38x_{1}+25.65x_{2}+0.07x_{3}+0.02x_{4}+10.65x_{5}+192.24x_{6}-436.38$$

(6) $$y_{2}=1.49x_{1}+24.29x_{2}+0.09x_{3}+0.02x_{4}+9.27x_{5}+168.30x_{6}-417.86$$

(7) $$y_{3}=1.25x_{1}+21.95x_{2}+0.07x_{3}+0.07x_{4}+11.21x_{5}+142.14x_{6}-379.82$$

(8) $$y_{4}=1.03x_{1}+6.16x_{2}+0.06x_{3}+0.09x_{4}+13.25x_{5}+160.58x_{6}-393.43$$

where *y*_1_, *y*_2_, *y*_3_, and *y*_4_ represent the calculated values of the four origin models of Yanchi County, Otoke Qianqi, Inner Mongolia, Dingbian County, Shaanxi, and Huanxian County, Gansu, respectively. Meanwhile, *x*_1_ to *x*_6_ represent the mineral element values (mg/kg) for ^111^Cd, ^52^Cr, ^56^Fe, ^23^Na, ^121^Sb, and ^78^Se. Using this discriminant model, the liver samples of Tan sheep were classified. The measured mineral element content value was brought into the above model to calculate the corresponding *y* value; the *y* value was compared, and then the samples were classified as the largest category of the *y* value. Finally, combined with the return-generation examination and hand-over-fork examination results, the validity of the established discriminant model was verified. The result data are shown in Table 10, and the discriminant figure is shown in Figure 5.

From Table 10, it can be seen that the overall correct discrimination rate of the return-generation and hand-over-fork examinations of the liver samples reached more than 85.00%, indicating a superior overall discriminatory effect. In both testing methods, the discrimination effect of each region showed minimal difference. Samples from each region were identified as being similar with those from other regions, possibly due to the origin of the Tan sheep being closer to these regions and having a similar growth environment; thus, this results in slight differentiation errors. The correct discrimination rate mentioned above exceeded 85.00%, indicating the effectiveness of the screened traceability indices in the discrimination of the origin of visceral tissue samples from the random Tan sheep. The selected elemental indicators serve as ideal information indicators for tracing the origin of the Tan sheep visceral tissues.

## 4. Discussion

In our study, the mineral content in the viscera of Tan sheep mostly differed among regions,. Following ANOVA analysis, with the exception of ^43^Ca, ^44^Ca, and ^24^Mg, the macronutrients including ^39^K, ^23^Na, and ^31^P differed in the heart and liver of Tan sheep in different regions, and the contents of ^39^K and ^23^Na were the highest in the heart and liver tissues of Tan sheep in Yanchi County, Ningxia (refer to Table 3 and Table 4). The trace elements ^137^Ba, ^56^Fe, and ^78^Se exhibited significant variations in the heart and liver samples of Tan sheep in different regions. Notably, the Tan sheep of Yanchi County in Ningxia had the highest levels of ^78^Se in both organs (refer to Table 3 and Table 4). The trace minerals ^9^Be, ^59^Co, ^133^Cs, ^95^Mo, ^85^Rb, ^118^Sn, and ^51^V were not significantly different in the heart and liver of Tan sheep in different regions (refer to Table 3 and Table 4), among which the contents of ^133^Cs, ^59^Co, and ^51^V were basically the same in the heart tissues from the four regions (refer to Table 3). The coefficient of variation is the ratio of the mean value to the standard deviation, which can reflect the degree of dispersion of the data. The larger the value, the greater the degree of dispersion and vice versa, and the smaller the coefficient of variation. The large coefficient of variation among regions may obscure the differences in mineral element contents, resulting in their insignificance when comparing regions.

Macro mineral elements play a very important role in maintaining the osmotic pressure of tissues and cells and the permeability of cell membranes by regulating the acid–base balance of body fluids and maintaining normal nerve conduction. Wang et al. [28] used ICP-MS to analyze 25 mineral elements in Tan sheep bones from various regions (Otoke Qianqi, Inner Mongolia; Yanchi County, Ningxia; Huanxian County, Gansu; and Dingbian County, Shaanxi). They found that the content of P was significantly higher in Yanchi County than in other regions, and that this result was consistent with the higher P content observed in the liver. Micronutrients are rarely found in animals, but they play a very important role in maintaining their growth and development. Animals mainly rely on plants to obtain micronutrients from the soil, so micronutrient deficiencies in feed can affect the nutrition and health of animals [21,29]. Wang, Liu, Zhao, Qie, Bai, Zhang, Guo, and Zhao [23] determined the mineral element contents of the meat from 104 sheep and 24 goats from different regions of Inner Mongolia, China. The findings revealed significant regional variations in the levels of eleven elements (Mg, Al, K, Ca, Mn, Fe, Cu, Zn, Rb, Sr, and Ba) present in lamb meat. Sun et al. [30] used ICP-MS to investigate the mineral element fingerprints of lamb samples from three pastoral areas in Alaska, Xilin Goleng, and Hulunbuir and two agricultural areas in Chongqing and Heze. The results showed that the elemental contents of the agricultural samples were generally higher than those of the pastoral samples. Moreover, nine elements including Ca, Zn, Be, Ni, Fe, Ba, Sb, Mn, and Se were found to be associated with local soils. The findings of the aforementioned studies collectively suggest that geographic location exerts an influence on the mineral element contents in lamb samples, and these results are congruent with those obtained from this experiment.

Soil texture is a major factor affecting the distribution and characteristics of mineral elements in the meat of Tan sheep. The soil texture in the north central area of Yanchi County, Ningxia, and the around Mengcheng is predominantly gray calcium soil, while the Loess Plateau hilly area features mainly black kiln soil. The northern gentle slope hilly area, on the other hand, is characterized by wind-sand soil. Based on the geomorphological feature and Tan sheep feeding patterns, three sampling sites (Mahuangshan Township, Fengji Gou Township, and Gaoshawo Township) were selected to comprehensively collect fingerprint information for Yanchi County. Furthermore, the distribution characteristics of mineral elements in the heart and liver tissues of Ningxia Yanchi Tan sheep were analyzed.

The contents of the mineral elements ^137^Ba, ^43^Ca, ^111^Cd, ^56^Fe, ^39^K, ^55^Mn, ^78^Se, ^125^Te, and ^66^Zn were significantly different in the heart and liver of Tan sheep from Yanchi, Ningxia (refer to Table 5 and Table 6), and the characteristic elements of heart and liver were mostly consistent among the four regions, among which the elements with the same characteristics were ^56^Fe and ^78^Se (refer to Table 5 and Table 6). Wang et al. [31] used ICP-MS to determine the mineral elements in the soils of Yanchi County, revealing significant differences in Fe content across Mahuangshan Township, Fengji Gou Township, and Gaoshawo Township. This finding further underscores the profound impact of geographical location on mineral element levels in Tan sheep. In the present experiment, liver samples of Yanchi Tan sheep exhibited a significantly higher Na concentration at 3299.25 mg/kg compared to other regions. The mineral element contents of Australian Merino, Damara, and Dorper sheep were assessed by ICP-MS, and the results indicated that the Na content in the control group for each breed was 2284.91 mg/kg, 2172.80 mg/kg, and 1983.91 mg/kg, respectively [32]. In comparison, Yanchi Tan sheep have a greater ability to accumulate Na, which can be attributed to the prevalence of saline soils in Yanchi County that are rich in NaCl and Na_2_CO_3_, resulting in a high deposition of sodium elements within the Tan sheep.

In order to further analyze the characteristic mineral elements in the visceral tissues of Yanchi County Tan sheep, PCA, HCA, and LDA were conducted so as to screen out the origin traceability indices of the heart and liver of Yanchi Tan sheep and to establish a discriminant model and validate it. In Tan sheep heart samples, the LDA results indicated an 85.70% accuracy rate for the return-generation examination and an 87.50% accuracy rate for the hand-over-fork examination. In Tan sheep liver samples, the LDA results indicated a 96.30% accuracy rate for the return-generation examination and an 86.25% accuracy rate for the hand-over-fork examination.

In the present study, the overall accuracy rate for distinguishing heart samples based on traceable fingerprints of mineral elements was above 85.00%. However, this result was lower than that reported by Sun, Guo, and Wei [16], as well as that reported by Zhang et al. [33]. This discrepancy may be attributed to various factors such as soil composition, climate conditions, and forage feeding practices that can influence mineral element content levels and consequently affect discrimination outcomes [34,35]. In the future, we plan to incorporate climate and soil factors, increase our sample size, and utilize stable isotopes and chemometric methods for regional representation to further enhance our fingerprint information and improve the accuracy in distinguishing Tan sheep offal from different origins. This will provide a theoretical basis for discriminating the origin of Tan sheep offal.

## 5. Conclusions

This study has demonstrated significant variations in the mineral element contents of heart and liver samples of Tan sheep from different regions in northwest China. The application of multivariate statistical analysis techniques (PCA, HCA, and LDA) yielded robust results on the discrimination of the geographical origin of these viscera samples. The results indicate that the multi-element analysis method was capable of distinguishing the heart and liver samples of Tan sheep from different geographical regions, and that it was able to identify elements that contribute to their regional characteristics. However, this study also had aspects that need to be improved, such as the limited sample size. In subsequent studies, the sample size should be increased, environmental factors such as climate and soil should be added, and a reliable classification model should be established to further improve the discrimination ability of the technique.

## Figures and Tables

**Table 1:** Information on visceral tissue samples of Tan sheep.

Region		Number		Altitude (m)	East Longitude	North Latitude
		Hearts	Livers			
Ningxia	Mahuangshan	10	10	1700	107.40	37.78
	Gaoshawo	10	10			
	Fengjiigou	10	10			
Inner Mongolia		10	10	1300	107.48	38.18
Shaanxi		10	10	1300	107.60	37.58
Gansu		10	10	1800	107.30	36.58

**Table 2:** Linear regression equations, correlation coefficients, RSDs, and LODs for the various elements.

Element	Various Element Regression Equations	Correlation Coefficients/r	RSD/%	LOD/(μg/L)
^107^Ag	*y* = 0.0057*x* + 0.0102	0.9991	4.4700	0.0050
^137^Ba	*y* = 0.4829*x* + 0.5407	0.9996	3.5200	0.0220
^9^Be	*y* = 0.0902*x* + 0.0025	0.9996	4.1900	0.0013
^43^Ca	*y* = 3.0085*x* + 7.3018	0.9990	6.9100	5.3420
^44^Ca	*y* = 0.0028*x* + 0.0139	0.9998	7.8000	1.3380
^111^Cd	*y* = 0.1029*x* + 2.4892	0.9999	5.6700	0.0055
^59^Co	*y* = 0.7839*x* + 0.0054	0.9979	6.7800	0.0017
^63^Cu	*y* = 0.2891*x* + 0.0000	0.9997	3.7600	0.0200
^52^Cr	*y* = 0.0382*x* + 0.0882	0.9997	5.8900	0.1390
^133^Cs	*y* = 3.0932*x* + 0.3298	0.9988	4.5400	0.0013
^56^Fe	*y* = 1.0936*x* + 0.9012	0.9999	4.6600	0.8185
^39^K	*y* = 0.1682*x* + 0.9720	0.9999	5.3700	2.2780
^24^Mg	*y* = 0.0190*x* + 0.1092	0.9995	2.9800	0.8306
^55^Mn	*y* = 0. 1092*x* + 0.9210	0.9995	2.8000	0.3609
^95^Mo	*y* = 0.2012*x* + 3.1754	0.9990	4.5000	0.0062
^23^Na	*y* = 1.0922*x* + 0.1192	1.0000	1.9000	0.1187
^60^Ni	*y* = 0.0021*x* + 0.0104	0.9989	5.6700	0.0269
^31^P	*y* = 0.0003*x* + 0.0183	0.9979	6.8700	3.3060
^85^Rb	*y* = 1.0032*x* + 10.7201	0.9999	4.6300	0.0198
^121^Sb	*y* = 0.2891*x* + 3.9023	0.9979	6.4300	0.2011
^78^Se	*y* = 0.0382*x* + 1.3280	0.9999	1.5900	0.0113
^88^Sr	*y* = 0.2011*x* + 3.1093	0.9990	3.9600	0.0911
^118^Sn	*y* = 0.3022*x* + 0.1046	0.9990	4.8400	0.0036
^125^Te	*y* = 0.4724*x* + 0.2093	0.9999	4.0800	0.0072
^51^V	*y* = 1.0321*x* + 0.0309	1.0000	1.7800	0.0102
^66^Zn	*y* = 0.4293*x* + 0.2389	0.9990	5.5900	0.2524

**Table 3:** Contents of 25 elements in lamb heart samples from different regions (mg/kg).

Element	Yanchi County of Ningxia	Otoke Qianqi of Inner Mongolia	Dingbian of Shaanxi Province	Huanxian County of Gansu Province
^137^Ba	0.13 ± 0.09 ^b^	0.18 ± 0.07 ^a^	0.05 ± 0.06 ^d^	0.11 ± 0.08 ^c^
^9^Be	0.01 ± 0.02 ^a^	0.03 ± 0.02 ^a^	0.03 ± 0.02 ^a^	0.02 ± 0.04 ^a^
^43^Ca	425.56 ± 20.37 ^b^	429.57 ± 37.89 ^b^	459.83 ± 27.78 ^a^	389.04 ± 14.68 ^c^
^44^Ca	401.23 ± 16.54 ^a^	404.43 ± 27.90 ^a^	415.85 ± 30.59 ^a^	399.20 ± 39.84 ^a^
^111^Cd	9.40 ± 0.58 ^a^	8.78 ± 2.95 ^a^	9.54 ± 2.78 ^a^	7.77 ± 2.10 ^a^
^59^Co	0.04 ± 0.05 ^a^	0.06 ± 0.02 ^a^	0.04 ± 0.04 ^a^	0.04 ± 0.00 ^a^
^63^Cu	16.78 ± 5.05 ^c^	24.89 ± 1.30 ^a^	19.75 ± 1.00 ^b^	20.42 ± 5.50 ^b^
^52^Cr	0.67 ± 0.31 ^a^	0.47 ± 0.15 ^a^	0.49 ± 0.18 ^a^	0.59 ± 0.18 ^a^
^133^Cs	0.08 ± 0.04 ^a^	0.07 ± 0.02 ^a^	0.06 ± 0.02 ^a^	0.08 ± 0.02 ^a^
^56^Fe	145.75 ± 12.47 ^d^	247.98 ± 28.32 ^b^	189.57 ± 18.39 ^c^	298.95 ± 21.10 ^a^
^39^K	16,518.90 ± 144.78 ^a^	13,209.84 ± 137.83 ^b^	10,399.03 ± 129.78 ^c^	15,980.06 ± 412.89 ^a^
^24^Mg	1202.95 ± 211.05 ^a^	1093.90 ± 176.34 ^a^	993.32 ± 236.90 ^a^	1190.45 ± 298.94 ^a^
^55^Mn	2.13 ± 0.28 ^a^	2.67 ± 0.78 ^a^	2.90 ± 0.55 ^a^	3.14 ± 1.55 ^a^
^95^Mo	0.23 ± 0.42 ^a^	0.28 ± 0.14 ^a^	0.14 ± 0.12 ^a^	0.31 ± 0.18 ^a^
^23^Na	5387.85 ± 467.38 ^a^	3980.04 ± 328.58 ^c^	5033.24 ± 173.53 ^a^	4786.09 ± 179.55 ^b^
^60^Ni	1.71 ± 0.34 ^a^	1.43 ± 0.16 ^ab^	1.67 ± 0.18 ^a^	1.23 ± 0.84 ^b^
^31^P	899.85 ± 48.99 ^b^	897.23 ± 34.34 ^b^	678.57 ± 21.34 ^c^	1029.78 ± 39.44 ^a^
^85^Rb	15.53 ± 3.05 ^a^	15.99 ± 11.23 ^a^	16.32 ± 2.09 ^a^	10.43 ± 3.48 ^a^
^121^Sb	10.43 ± 4.31 ^a^	11.43 ± 1.89 ^a^	13.98 ± 4.33 ^a^	13.38 ± 2.66 ^a^
^78^Se	0.52 ± 0.14 ^a^	0.33 ± 0.04 ^b^	0.19 ± 0.08 ^c^	0.47 ± 0.12 ^a^
^88^Sr	1.62 ± 0.48 ^a^	2.05 ± 0.18 ^a^	1.98 ± 0.44 ^a^	1.46 ± 0.68 ^a^
^118^Sn	0.33 ± 0.43 ^a^	0.29 ± 0.14 ^a^	0.32 ± 0.28 ^a^	0.30 ± 0.04 ^a^
^125^Te	4.01 ± 2.21 ^a^	1.98 ± 0.26 ^c^	2.54 ± 0.88 ^b^	3.67 ± 0.44 ^ab^
^51^V	0.07 ± 0.12 ^a^	0.08 ± 0.02 ^a^	0.07 ± 0.02 ^a^	0.08 ± 0.02 ^a^
^66^Zn	97.90 ± 13.78 ^a^	88.54 ± 10.04 ^b^	67.65 ± 19.32 ^c^	60.34 ± 19.05 ^c^

Values in rows followed by different letters are significantly different (*p* < 0.05).

**Table 4:** Contents of 25 elements in lamb liver samples from different regions (mg/kg).

Element	Yanchi County of Ningxia	Otoke Qianqi of Inner Mongolia	Dingbian of Shaanxi Province	Huanxian County of Gansu Province
^107^Ag	0.02 ± 0.00 ^a^	— ^a^	0.01 ± 0.00 ^a^	0.02 ± 0.00 ^a^
^137^Ba	0.78 ± 0.08 ^c^	0.99 ± 0.12 ^b^	1.02 ± 0.08 ^b^	1.29 ± 0.84 ^a^
^9^Be	0.00 ± 0.00 ^a^	0.02 ± 0.02 ^a^	0.01 ± 0.00 ^a^	0.01 ± 0.00 ^a^
^43^Ca	324.53 ± 19.88 ^a^	298.90 ± 82.12 ^a^	340.85 ± 14.78 ^a^	330.03 ± 28.34 ^a^
^44^Ca	300.43 ± 25.54 ^a^	288.98 ± 33.66 ^a^	302.43 ± 20.85 ^a^	295.57 ± 19.90 ^a^
^111^Cd	267.86 ± 11.82 ^b^	300.97 ± 19.48 ^a^	235.64 ± 29.89 ^bc^	198.97 ± 20.26 ^c^
^59^Co	0.11 ± 0.18 ^a^	0.25 ± 0.32 ^a^	0.14 ± 0.02 ^a^	0.23 ± 0.04 ^a^
^63^Cu	109.90 ± 9.93 ^a^	89.98 ± 10.39 ^a^	113.06 ± 22.22 ^a^	114.03 ± 9.53 ^a^
^52^Cr	0.11 ± 0.04 ^c^	0.53 ± 0.15 ^a^	0.39 ± 0.14 ^b^	0.34 ± 0.22 ^b^
^133^Cs	0.01 ± 0.00 ^a^	0.02 ± 0.01 ^a^	0.04 ± 0.04 ^a^	0.03 ± 0.02 ^a^
^56^Fe	524.64 ± 78.63 ^b^	478.90 ± 28.42 ^c^	669.43 ± 78.32 ^a^	701.03 ± 89.43 ^a^
^39^K	10,983.05 ± 821.89 ^a^	10,055.69 ± 278.04 ^a^	8865.83 ± 329.89 ^b^	10,544.35 ± 390.05 ^a^
^24^Mg	998.67 ± 89.22 ^a^	897.84 ± 11.32 ^b^	704.34 ± 90.32 ^c^	893.39 ± 67.54 ^b^
^55^Mn	15.12 ± 1.89 ^b^	12.65 ± 1.88 ^b^	9.42 ± 1.89 ^c^	17.98 ± 3.56 ^a^
^95^Mo	3.96 ± 2.67 ^a^	4.72 ± 1.58 ^a^	4.39 ± 0.68 ^a^	4.89 ± 1.24 ^a^
^23^Na	3299.25 ± 328.73 ^a^	2490.87 ± 289.08 ^b^	2383.47 ± 231.87 ^b^	2904.49 ± 521.03 ^a^
^60^Ni	1.22 ± 0.99 ^a^	0.67 ± 0.55 ^a^	0.89 ± 0.22 ^a^	0.98 ± 0.45 ^a^
^31^P	2430.36 ± 232.98 ^a^	1874.96 ± 127.54 ^b^	1290.49 ± 98.94 ^c^	1689.39 ± 167.04 ^b^
^85^Rb	18.32 ± 3.83 ^a^	14.23 ± 2.25 ^a^	16.89 ± 4.32 ^a^	13.02 ± 3.55 ^a^
^121^Sb	21.32 ± 8.22 ^b^	18.32 ± 5.33 ^b^	27.43 ± 1.24 ^a^	30.21 ± 2.88 ^a^
^78^Se	0.66 ± 0.44 ^a^	0.32 ± 0.08 ^b^	0.11 ± 0.00^d^	0.22 ± 0.00 ^c^
^88^Sr	0.57 ± 0.18 ^c^	0.96 ± 0.06 ^a^	0.79 ± 0.04 ^b^	1.23 ± 0.66 ^a^
^118^Sn	0.02 ± 0.01 ^a^	0.01 ± 0.01 ^a^	0.01 ± 0.00 ^a^	0.03 ± 0.04 ^a^
^125^Te	0.19 ± 0.18 ^a^	0.28 ± 0.45 ^a^	0.24 ± 0.04 ^a^	0.26 ± 0.04 ^a^
^51^V	0.04 ± 0.04 ^a^	0.05 ± 0.10 ^a^	0.06 ± 0.00 ^a^	0.07 ± 0.03 ^a^
^66^Zn	168.75 ± 32.21 ^a^	153.46 ± 12.48 ^a^	169.83 ± 34.55 ^a^	149.36 ± 23.32 ^a^

Values in rows followed by different letters are significantly different (*p* < 0.05).

**Table 5:** Contents of 13 elements in lamb heart samples from Yanchi regions (mg/kg).

Element	Mahuangshan Township	Gaoshawo Township	Fengjigou Township
^137^Ba	0.17 ± 0.04 ^a^	0.08 ± 0.00 ^c^	0.13 ± 0.05 ^b^
^43^Ca	475.70 ± 29.78 ^a^	393.47 ± 34.73 ^b^	407.38 ± 32.28 ^b^
^111^Cd	12.40 ± 2.89 ^a^	6.90 ± 0.43 ^b^	8.90 ± 0.49 ^b^
^63^Cu	18.73 ± 3.77 ^a^	20.78 ± 3.42 ^a^	10.53 ± 2.32 ^b^
^56^Fe	127.34 ± 9.48 ^c^	140.96 ± 23.27 ^b^	168.20 ± 28.20 ^a^
^39^K	18,932.48 ± 498.13 ^a^	15,306.13 ± 893.57 ^b^	12,892.90 ± 748.74 ^c^
^55^Mn	1.93 ± 0.33 ^b^	2.02 ± 0.43 ^b^	2.44 ± 0.55 ^a^
^60^Ni	1.92 ± 0.67 ^a^	1.53 ± 0.33 ^a^	0.69 ± 0.05 ^b^
^31^P	930.23 ± 78.37 ^a^	823.32 ± 66.28 ^b^	943.81 ± 53.25 ^a^
^78^Se	0.42 ± 0.33 ^b^	0.48 ± 0.56 ^a^	0.34 ± 0.01 ^c^
^118^Sn	0.53 ± 0.08 ^a^	0.13 ± 0.02 ^c^	0.33 ± 0.00 ^b^
^125^Te	3.95 ± 0.67 ^b^	4.04 ± 1.02 ^a^	3.99 ± 0.54 ^a^
^66^Zn	86.34 ± 12.66 ^b^	99.28 ± 12.45 ^b^	108.08 ± 53.23 ^a^

Values in rows followed by different letters are significantly different (*p* < 0.05).

**Table 6:** Contents of 16 elements in lamb liver samples from Yanchi regions (mg/kg).

Element	Mahuangshan Township	Gaoshawo Town	Fengjigou Township
^137^Ba	0.95 ± 0.03 ^a^	1.03 ± 0.08 ^a^	0.36 ± 0.04 ^b^
^43^Ca	386.01 ± 21.28 ^a^	300.31 ± 45.34 ^b^	288.32 ± 29.67 ^b^
^44^Ca	354.34 ± 13.45 ^a^	301.34 ± 20.43 ^b^	298.48 ± 15.39 ^b^
^111^Cd	289.32 ± 44.76 ^a^	247.32 ± 34.38 ^c^	265.05 ± 12.27 ^b^
^59^Co	0.11 ± 0.08 ^a^	0.18 ± 0.08 ^a^	0.03 ± 0.00 ^b^
^52^Cr	0.13 ± 0.00 ^a^	0.06 ± 0.00 ^b^	0.14 ± 0.00 ^a^
^56^Fe	592.12 ± 56.73 ^a^	477.32 ± 34.39 ^c^	503.91 ± 67.93 ^b^
^39^K	11,892.29 ± 1355.48 ^a^	10,289.27 ± 1243.06 ^b^	10,768.28 ± 1329.75 ^b^
^55^Mn	13.98 ± 6.56 ^b^	10.54 ± 2.75 ^c^	20.84 ± 4.45 ^a^
^95^Mo	4.32 ± 0.66 ^a^	3.28 ± 0.35 ^b^	4.28 ± 3.64 ^a^
^23^Na	3478.23 ± 39.83 ^a^	3002.87 ± 54.59 ^b^	3417.22 ± 46.10 ^a^
^121^Sb	20.58 ± 2.65 ^b^	23.45 ± 4.44 ^a^	19.93 ± 2.43 ^c^
^78^Se	0.73 ± 0.12 ^c^	0.84 ± 0.05 ^b^	0.91 ± 0.04 ^a^
^88^Sr	0.63 ± 0.04 ^a^	0.48 ± 0.22 ^b^	0.60 ± 0.00 ^a^
^125^Te	0.21 ± 0.04 ^a^	0.24 ± 0.02 ^a^	0.12 ± 0.04 ^b^
^66^Zn	175.33 ± 26.84 ^a^	169.34 ± 20.03 ^b^	160.62 ± 17.75 ^c^

Values in rows followed by different letters are significantly different (*p* < 0.05).

**Table 7:** Fisher discriminant function coefficient.

Element	Yanchi County of Ningxia	Otoke Qianqi of Inner Mongolia	Dingbian of Shaanxi Province	Huanxian County of Gansu Province
^43^Ca	0.17	0.13	0.18	0.14
^63^Cu	−0.97	0.02	0.27	−0.08
^39^K	0.09	0.08	0.05	0.07
^31^P	0.11	0.12	0.08	0.12
^78^Se	33.05	43.42	44.80	32.36
^125^Te	−0.90	0.05	−1.31	−1.09
^66^Zn	0.14	0.14	0.29	0.17
Constant	−161.79	−158.22	−124.86	−144.56

**Table 8:** LDA results of Tan sheep heart samples from different regions.

		Category of Origin	Forecast Category				Overall
			Otoke Qianqi of Inner Mongolia	Yanchi County of Ningxia	Huanxian County of Gansu Province	Dingbian of Shaanxi Province	
Return -generation examination	number	Otoke Qianqi of Inner Mongolia	38	2	0	0	40
		Yanchi County of Ningxia	0	35	2	3	40
		Huanxian County of Gansu Province	2	2	36	0	40
		Dingbian of Shaanxi Province	4	6	2	28	40
	Accuracy rate (%)		95.00	87.50	90.00	70.00	85.70
Hand-over -fork examination	number	Otoke Qianqi of Inner Mongolia	35	1	2	2	40
		Yanchi County of Ningxia	0	38	1	1	40
		Huanxian County of Gansu Province	3	0	35	2	40
		Dingbian of Shaanxi Province	4	0	4	32	40
	Accuracy rate (%)		87.50	95.00	87.50	80.00	87.50

**Table 9:** Fisher discriminant function coefficient.

Element	Yanchi County of Ningxia	Otoke Qianqi of Inner Mongolia	Dingbian of Shaanxi Province	Huanxian County of Gansu Province
^111^Cd	1.38	1.49	1.25	1.03
^52^Cr	25.65	24.29	21.95	6.16
^56^Fe	0.07	0.09	0.07	0.06
^23^Na	0.02	0.02	0.02	0.09
^121^Sb	10.65	9.27	11.21	13.25
^78^Se	192.24	168.30	142.14	160.58
constant	−436.38	−417.86	−379.82	−393.43

**Table 10:** LDA results of Tan sheep liver samples from different regions.

		Category of Origin	Forecast Category				Overall
			Otoke Qianqi of Inner Mongolia	Yanchi County of Ningxia	Huanxian County of Gansu Province	Dingbian of Shaanxi Province	
Return -generation examination	number	Otoke Qianqi of Inner Mongolia	36	4	0	0	40
		Yanchi County of Ningxia	0	40	0	0	40
		Huanxian County of Gansu Province	0	0	40	0	40
		Dingbian of Shaanxi Province	0	0	2	38	40
	Accuracy rate (%)		90.00	100.00	100.00	95.00	96.30
Hand-over -fork examination	number	Otoke Qianqi of Inner Mongolia	32	1	3	4	40
		Yanchi County of Ningxia	1	36	2	1	40
		Huanxian County of Gansu Province	2	1	34	3	40
		Dingbian of Shaanxi Province	2	0	2	36	40
	Accuracy rate (%)		80.00	90.00	85.00	90.00	86.25

## Data Availability

Data are only available upon request due to privacy and ethical restrictions. The data presented in this study are available upon request from the corresponding author. The data are not publicly available due to all data in this manuscript being of a confidential commercial nature.
